# Association of Serum Leptin Level with Obesity in Children with Acute Lymphoblastic Leukemia 

**Published:** 2015-07-20

**Authors:** S Zareifar, S Shorafa, S Haghpanah, Z Karamizadeh, R Adelian

**Affiliations:** 1 Hematology Research Center,Pediatric Hematology/Oncology department, Shiraz University of Medical Sciences, Shiraz, Iran.; 2 Department of Pediatrics, Shiraz University of Medical Sciences, Shiraz, Iran.; 3 Division of Pediatric Endocrinology, department of Pediatrics, Shiraz University of Medical sciences, Shiraz, Iran.

**Keywords:** Acute Lymphoblastic Leukemia, BMI, Leptin, Obesity

## Abstract

**Background:**

Obesity is a medical problem in survivors of childhood acute lymphoblastic leukemia. Obesity is associated with many complications, so it is important to investigate the respective etiology. Leptin is a protein synthesized in the fatty tissue and is effective in the control of obesity. Survey of leptin in acute lymphoblastic leukemia (ALL) survivors could be helpful in controlling obesity.

**Materials and Methods:**

In this prospective study, 53 pediatric patients diagnosed with ALL between 2006 and 2012 from Southern Iran, were enrolled. We examined body mass index (BMI) status and performed laboratory measuring tests including triglyceride, cholesterol, fasting blood sugar, leptin at diagnosis time and then every 6 months and in the last visit.

**Results:**

Participants consisted of 35 male and 18 female patients. At the time of diagnosis, 5.66% were overweight or obese, whereas at the end of treatment, approximately 13 patients (24.53%) were overweight or obese. The median and interquartile range (IQR) for blood leptin level were significantly higher for obese patients than other patients (885, 1120 vs. 246, 494 pg/ml), (P=0.030). The median and IQR were also significantly higher in females than in males (861, 969 vs. 204, 267 pg/ml), (P=0.006).

**Conclusion:**

Obesity is a complication of ALL treatment. It is associated with elevated blood leptin level. Hypothalamus leptin resistance in obese patients should be considered. In each visit, clinicians should weight and their patient’s BMI take into account.

## Introduction

The annual incidence of acute lymphoblastic leukemia (ALL), especially from 1975 to 2006, has increased 0.8% per year (1). Medical late effects of ALL, as the most common childhood cancer associated with cure rate of 80%, are noticeable. One or more adverse events occur in 74.1% of the acute leukemia survivors. Defective physical growth, especially obesity was most commonly reported (2). Obesity is increasingly prevalent in developed societies with considerable morbidity. This is especially important in children with ALL, in whom the metabolic syndrome may begin during therapy, reaching 40% at the end of treatment (3). In different studies, the prevalence of obesity has been reported to be16 to 57 percent among cured patients with ALL (4). Obesity at diagnosis has been even associated with disease prognosis (5,6). Several mechanisms intervene in the development of obesity in children with ALL. During treatment, patients usually undergo changes in their lifestyles. The loss of physical activity may be due to many factors, including diminished interest in recreational activity and 

over-protection of the child by parents or caregivers, changes in lifestyle7, diminished exercise capacity, impaired motor function because of steroid-related myopathy, and vincristine-related neuropathy (1,7). Reduced habitual physical activity and over-feeding due to corticosteroid therapy are commonly considered as the main factors responsible for weight gain. Leptin secretion impairment3, cranial radiotherapy, growth hormone insufficiency (4, 8-10) and pathophysiological changes in the cardiorespiratory system7 might also contribute to reduce physical activity leading to obesity. 

Adipocyte-derived hormone leptin, known as the prototypical adipokine, is the product of the ob gene. It is produced primarily in the adipose tissue but is expressed in a variety of tissues including the placenta, ovaries, mammary epithelium, bone marrow, and lymphoid tissues (11). Leptin reduces the appetite naturally and increases the food intake and energy expenditure.

In addition to regulating the body weight, leptin also has been shown to play a regulatory role for stimulation and differentiation within the myeloid, erythroid; and monocyte cell lineages; whereas; the results of its regulatory effects on lymphocytes and related tumor cells have been contradictory (11). In this study, we assessed the leptin level in children with ALL who developed obesity and overweight compared to patients with normal ranged BMI. 

## Materials and Methods

In this cohort study, 53 pediatric patients with diagnosis of ALL between 2006 and 2012 were enrolled. From a total of 105 ALL pediatric patients referred to the Pediatric Oncology Hospital referral center in Shiraz Southern Iran, 90 patients agreed to take part in this study. The diagnosis of ALL was confirmed with bone marrow aspiration and biopsy, flowcytometery and immunohistochemistry (IHC). Patients younger than 2 years, secondary malignancy, Down syndrome, hypothyroidism, history of familial hyperlipidemia, and ALL relapse were excluded. Finally 53 patients completed the study period.

Ethics committee approval was obtained by the Research Advisory Council (RAC) at Shiraz University of Medical Sciences. Informed written consents were obtained from the participants.

All patients received prednisone, vincristine,and L-asparaginase as induction chemotherapy. In high risk patients, doxorubicine was also added. Most patients received dexamethasone, daunorubicin, doxorubicin, cyclophosphamide, cytarabine, thioguanine methotrexate, and mercaptopurine as consolidation and maintenance therapy. Drugs were dosed by body-surface area. All patients received CNS chemo-prophylaxis with age-adjusted dose of intra-thecal methotrexate. Cranial radiation was administered to most high-risk patients. There were no protocol-required treatment modifications for obesity. Individual reports from the obese patients in the study cohort have been reviewed to assess for off- protocol dose modifications as the result of obesity.

BMI percentile chart was calculated from the book 2000 CDC Growth Charts for the United States: Methods and Development. 

Those with a BMI below the 5th percentile of the normal population were considered low weight and BMI between the 5th to less than 85th percentile, as normal BMI. 

BMI between 85 to less than 95% was considered as overweight and those equal to or above the 95th percentile were classified as obese.

Ten ml fasting blood was collected; the sera were separated and stored at- 20 centigrade to check leptin, insulin, fasting blood sugar (FBS), and total cholesterol, low density lipoprotein (LDL)and high density lipoprotein (HDL) levels. Leptin level (Pg/ml) was measured using enzyme-linked immunosorbent assay (ELISA) method (leptin ELISA kit, Orgenium, Finland) and insulin was also measured by the same method (Monobind, USA).

**Statistical analysis**

Data were analyzed by SPSS, v.17. Test of normality was performed by Shapiro-Wilk test. Descriptive data were presented as mean and standard deviation in data with normal distribution. In case of non normal distribution, data were summerized as median and interquartile range (IQR). Comparison of qualitative data between two groups was done using Chi-square test. Quantitative data were compared by Student t-test and Mann-Whitney test as appropriate between two groups of patients. Correlation of leptin and insulin levels was assessed by Pearson Correlation test. Comparison of BMI of different occasions with baseline was done by Paired t-test. P value less than 0.05 was considered statistically significant.

## Results

Thirty five (66%) patients were male and 18 (34%) female. The mean age at diagnosis was 6 ±3.9 years, ranged from 2 to16.4 years and the average duration of the follow-up was 41.6 ± 18.6 months, ranged from 6 to 89 months. The mean age at the last follow-up was 9.45± 4.2 years (range: 2.8-18.8 years). Ten (18.9%) out of 53 ALL treated children received cranial radiotherapy, consisting of 8males and 2 females.The patients’ characteristics are shown in Table I.

Obesity and BMI: At the time of diagnosis, 94.34% of the patients were normal or underweight;whereas; 5.7% were overweight or obese. Meanwhile, at the last follow up, these rates reached 75.5% and 24.5%, respectively. The difference was statistically significant (P = 0.04). 

**Figure1:**
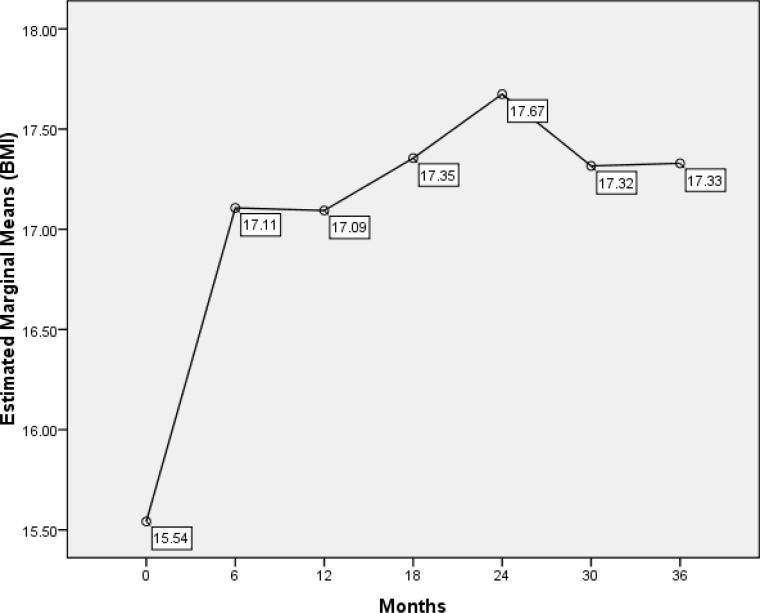
Changes in BMI at 3-year intervals from the time of diagnosis in ALL patients

Compared with BMI at diagnosis, increasing BMI was statistically significant after 6 months of starting treatment (15.5 vs. 17.1) (P< 0.001). (Fig1)

In the second 6 months after starting the treatment, BMI decreased slightly (P=0.02), but 2 years after treatment, it significantly increased (P=0.001). During 24 to 36 months after the diagnosis, BMI exhibited a decline that was not statistically significant (P=0.15).The BMI at diagnosis was significantly different from its increment at 6, 12, 18, 24, 30, and 36 months after treatment (P<0.05).The differences between male and female BMI indices at the last follow-up were not significant (P= 0.34).

Overally the median and IQR for leptin level were 265 and 771pg/ml( range:15- 3190 pg/ml). The mean and standard deviation was also 544±653 Pg / ml. Comparison of serum leptin, triglyceride, cholesterol and insulin levels as well as white blood cell of the two groups of patients with BMI <85% percentile and those with BMI ≥ 85% are summarized in Table II.

From all laboratory data evaluated, leptin, FBS, and total cholesterol showed significantly higher values in obese and overweight patients than those in patients with normal weight (P<0.05). The median for Leptin levels was higher in females than male patients, and the difference was statistically significant (861 (969) vs. 204 (267) pg/ml), (P= 0.006).

Although the fasting blood sugar only in one case was reported over 100 mg/dl, there were statistically significant differences between serum levels of fasting blood sugar of the obese and non-obese patients (82 mg /dl versus 88 mg/dl, P=0.029).

Aforementioned laboratory data and BMI were compared between the two groups of patients with and without cranial radiotherapy (Table III). Only TG and HDL cholestrol was significantly higher in patients with cranial radiotherapy, compared to patients without cranial radiotherapy (P=0.022 and P=0.041 respectively). Although median of serum leptin levels in patients who had received CRT was higher than those who didn’t (344 (861)pg/ml versus 253 (776) pg/ml), the difference was not statistically significant (P=0.426). 

**Figure 2:**
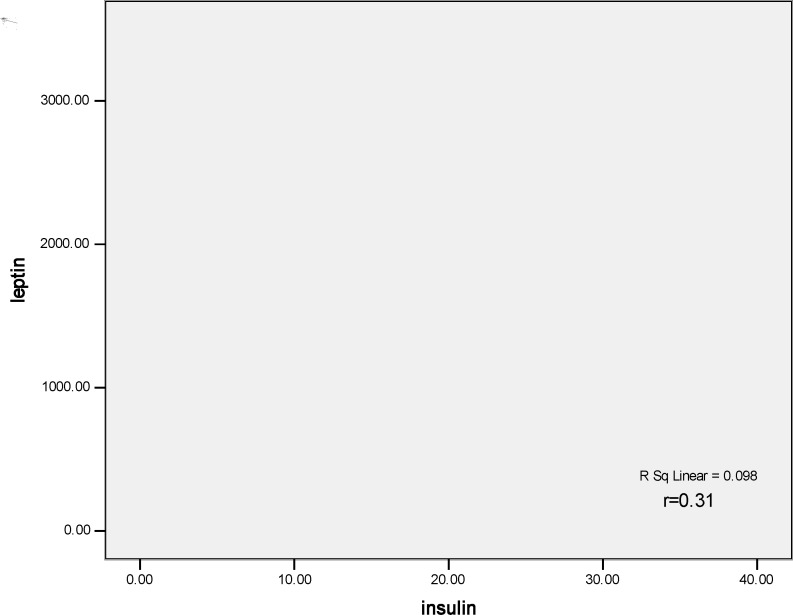
Correlation of insulin and serum leptin levels in ALL patients

There was no significant difference between BMI of the patients who had received CRT with those who had not (P = 0.484). There was also a positive statistically significant correlation between serum levels of insulin and leptin (r =0.31, P= 0.022) (Fig 2).

**Table I:** Demographic data of ALL patients

	**Female** **(n=18)**	**Male** **(n=35)**	**Total** **(n=53)**
Age at diagnosis(month)	71.94±46.47	71.94±46.47	**71.94±46.47**
Flow up time(month)	41.55±18.60	41.55±18.60	**41.55±18.60**
BMI≥85% percentile	5	8	**13**
CRT	2	8	**10**

**Table II:** Variables characteristics of ALL patients in terms of BMI

**Variable**	**BMI<85% percentile** **n=40**	**BMI≥85% percentile** **n=13**	**P-Value**
**Insulin(microunit/ml)** **Median (IQR)**	6.9 (6.2)	8.6 (6.8)	**0.092**
**FBS(mg/dl)** **Mean ± sd**	82 ± 8	88 ± 6.2	0.029*
**Insulin/glucose** **Median (IQR)**	0.085 (0.06)	0.097 (0.07)	**0.172**
**Leptin(p.gr/ml)** **Median (IQR)**	246 (494)	885 (1120)	0.030*
**Total Cholestrol(mg/dl)** **Mean ± sd**	150 ± 29	172 ± 32	0.032*
**LDL Cholestrol(mg/dl)** **Mean ± sd**	86 ± 27	98 ± 25	**0.165**
**HDL Cholestrol(mg/dl)** **Median (IQR)**	40 (13.4)	43.7 (19)	**0.657**
**TG(mg/dl)** **Median (IQR)**	116 (113)	109 (150)	**0.775**
**WBC/mm³ at diagnosis** **Median (IQR)**	10950 (32925)	4700 (22700)	**0.422**

* Statistically significant

**Table III:** Variable characteristics of ALL patients in terms of CRT

**Variable**	**Without CRT** **n=43**	**With CRT** **n=10**	**P-Value**
Insulin(microunit/ml) Median (IQR)	7.4(6.3)	7.7 (7.3)	**0.776**
FBS(mg/dl) Mean ± sd	84±7	83±8	**0.713**
Insulin/glucose Median (IQR)	0.088(0.06)	0.093( 0.08)	**0.682**
Leptin(p.gr/ml) Median (IQR)	253(776)	344(861)	**0.426**
Total Cholestrol(mg/dl) Mean ± sd	152±29	169±35	**0.125**
LDL Cholestrol(mg/dl) Mean ± sd	89±24	88±38	**0.958**
HDL Cholestrol(mg/dl) Median (IQR)	39(11)	49(17)	0.041*
TG(mg/dl) Median (IQR)	104(94)	164(190)	0.022*
BMI Median (IQR)	16(4.6)	16.3(3.6)	**0.484**

* Statistically significant

## Discussion

In the present study, 35 patients (66%) were males, representing a greater prevalence of childhood ALL in males (13). Obesity was evident in 5.66% of patients with ALL at the time of diagnosis and BMI significantly increased with treatment. It reflects the nutrition disturbance and weight loss in early stages of their diseases and may be the result of underlying disease. At the last follow-up, 24.53% of the patients were obese. 

The prevalence of obesity varies in different research. In the study by Zhang et al on 83 patients, the rates of obesity (BMI ≥ 85% percentile) at the time of diagnosis and 5 years after treatment were approximately 21% and 40%, respectively (9). 

Aldhafiri and colleagues reported 35% obesity at diagnosis (10). Jansen and colleagues reported obesity rates in 52% of patients with ALL and7.41 % of patients with brain tumor, 12 months after completion of the therapy (11). 

Some studies reported the incidence of obesity at the time of diagnosis and at the end of treatment 15% and 40%, respectively (12). Considering the rate of 20 percent obesity in healthy children (13, 14), there was no statistically difference between the rates of obesity before and after treatment and the rate in normal population.

 In a study, 2 years after starting treatment, there was no significant difference between normal population and patients with ALL (6). Six months after starting treatment, our study also showed a significant increase in BMI which is consistent with Zhang’s study (9). In general, two years after starting treatment, the BMI trend demonstrated an ascending pattern followed by slightly decreasing and again increasing with slower slope. 

High-dose corticosteroid therapy may be one of the reasons of sudden increase in weight in the first 6 months, which starts at the beginning of treatment. The high sensitivity of parents in their children's diet, excessive care, and limited physical activity may be important in respective patients’ weight gain in the early months of diagnosis.

Weight loss was shown in our patients during 30 months after treatment. Although this weight loss was not statistically significant, it should be considered clinically important in need of investigation. After that, a slow upward trend in BMI was evident. After an average follow-up of approximately 42 months, a significant increase in BMI was noticed.

Serum leptin levels in patients with BMI ≥ 85% showed a significant increase, compared to those with BMI <85% percentile. There was a significant difference in total cholesterol and fasting blood glucose in two groups, the differences in serum TG, LDL, HDL, and serum insulin were not significant. 

Mean leptin levels in patients under investigation were around 544 ± 653 Pg / ml. There was no control group in this study, but in a report from Iran, mean leptin level in healthy children was 9220 ± 2900 pg/ml^41^. According to some studies, normal leptin level in men with less than 15 percent body fat and women with less than 25 percent body fat have been reported 1000 - 16000Pg / ml, respectively (15,16). 

It is evident that level of leptin in patients with ALL is lower than normal counterparts. Theoretically, it could be because of impaired adipose tissue leptin production. 

Heike Wex et al revealed that in patients with ALL, the leptin level at the time of diagnosis was lower than the levels of healthy control subjects, but thirty-three days after the diagnosis it was more than that in normal controls (17). Haddy reported that leptin levels after treatment of patients with ALL are higher than that among normal people (2). It seems that leptin levels are various in different phases of ALL therapy; further studies are needed for better assessment of its variation and its association in response to treatment.

In the present study, comparison of serum leptin levels in obese and non-obese patients with ALL showed a significant increase.The girl also had higher serum leptin levels than boys, as demonstrated in some previous studies (10, 18-20). 

Unlike the majority of previous studies, patients who had received CRT were a little fatter and had higher leptin levels, the difference was not statistically significant; this is in agreement with Withycomb^21 ^and Razzouk (22) studies but in contrast with some other research (5,18).

Unlike some previous studies (4,9), there were no significant differences between serum levels of total cholesterol, LDL, insulin, and BMI values in the patients who had received CRT, and those who had not ​​. However, TG and HDL cholesterol were significantly different between two groups of patients. 

Regarding the rate of obesity, certain attention and screening are needed in patients with ALL during the first 6 months of treatment, because the BMI trend in this interval is very fast. Many factors may be involved in increasing BMI in children with ALL, including high-dose steroids (13), gender (12), CRT (18,5), physical activity (23), and resistance to leptin (6). Parents should care more about children’s nutrition; otherwise, rapid weight gain can occur in the community.

Based on our findings, obesity, as a highly morbid disease, should be considered in ALL survivors. 

Early treatment and monitoring of patients with ALL along with their timely management may prevent complications.

## Conclusion

Childhood cancer survivors require lifelong monitoring, with prompt identification and treatment of adverse late effects. In general, leptin, as a factor causing obesity, is affected by several factors such as steroid therapy, CRT, chemotherapy, and resistance to central leptin. Given these factors, it seems essential to control obesity In ALL survivors. 
